# Marked gallbladder wall thickening caused by Epstein–Barr virus‐induced infectious mononucleosis

**DOI:** 10.1002/ccr3.8863

**Published:** 2024-06-07

**Authors:** Masahiko Nakamura, Shun Yamashita, Masaki Tago, Shu‐ichi Yamashita

**Affiliations:** ^1^ Department of General Medicine Saga University Hospital Saga Japan; ^2^ Education and Research Center for Community Medicine, Faculty of Medicine Saga University Saga Japan

**Keywords:** acalculous cholecystitis, Epstein–Barr virus, gallbladder wall thickening, infectious mononucleosis

## Abstract

**Key Clinical Message:**

In patients with symptoms of viral infection and marked thickening of the gallbladder wall, it is important to suspect acalculous cholecystitis due to Epstein–Barr virus‐induced infectious mononucleosis.

**Abstract:**

A 35‐year‐old Japanese man presented with fever, abdominal right upper quadrant pain, and liver dysfunction. Positive immunoglobulin M and ‐G antibodies and negative nuclear antigen for Epstein–Barr virus were observed. Abdominal ultrasonography revealed a markedly thickened gallbladder wall. Acalculous cholecystitis due to Epstein–Barr virus‐induced infectious mononucleosis was diagnosed.

## Graphical abstract

Marked thickening of the gallbladder wall is important to suspect acalculous cholecystitis due to Epstein–Barr virus‐induced infectious mononucleosis.

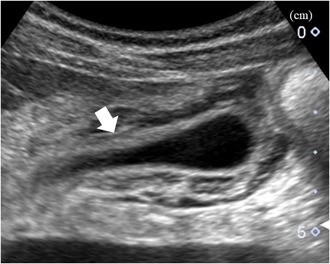

## 1 INTRODUCTION

Infectious mononucleosis (IM) due to Epstein–Barr virus (EBV) is rarely complicated with acalculous cholecystitis (AAC).^1^ We herein report a case of AAC due to EBV‐induced IM.

## 2 CASE PRESENTATION

**FIGURE 1:**
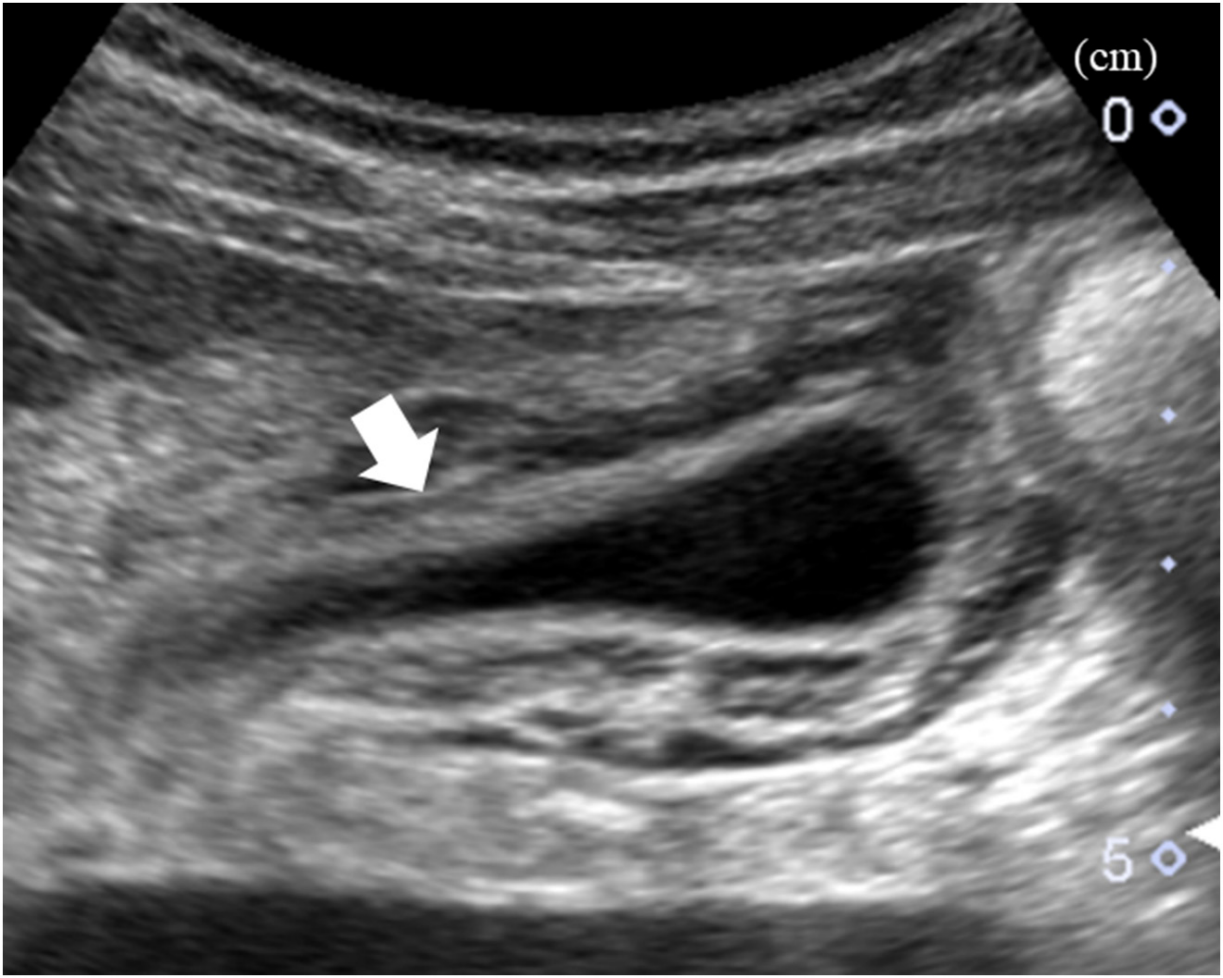
Findings of the gallbladder wall on abdominal ultrasonography on admission. Abdominal ultrasonography on admission shows the thickened gallbladder wall measuring 10 mm (arrow).

**FIGURE 2:**
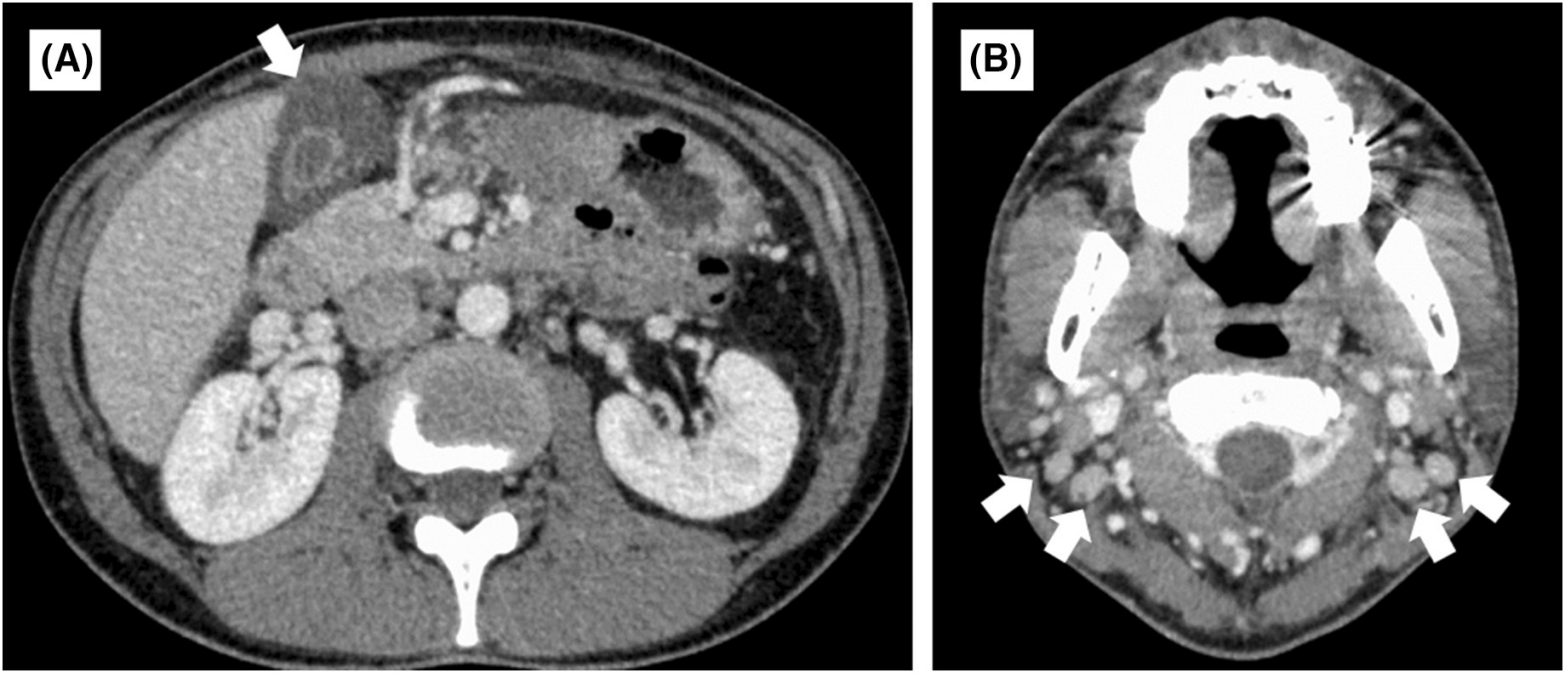
Findings of chest and abdominal computed tomography with contrast enhancement. Chest and abdominal computed tomography with contrast enhancement shows a markedly thickened gallbladder wall measuring 12 mm (A, arrow) and multiple enlarged cervical lymph nodes (B, arrows).

**FIGURE 3:**
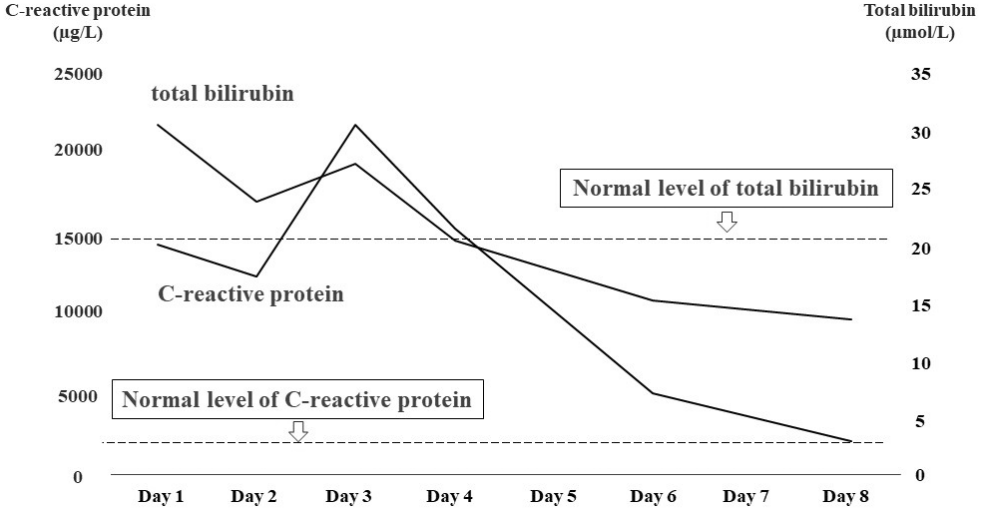
Evolution of C‐reactive protein and total bilirubin values. On admission, the C‐reactive protein value was 14,000 μg/L (reference range, 0–1400), and the total bilirubin value was 30.8 μmol/L (reference range, 6.8–20.5). These values improved gradually and normalized on Day 8 and 4 (C‐reactive protein and total bilirubin, respectively).

**FIGURE 4:**
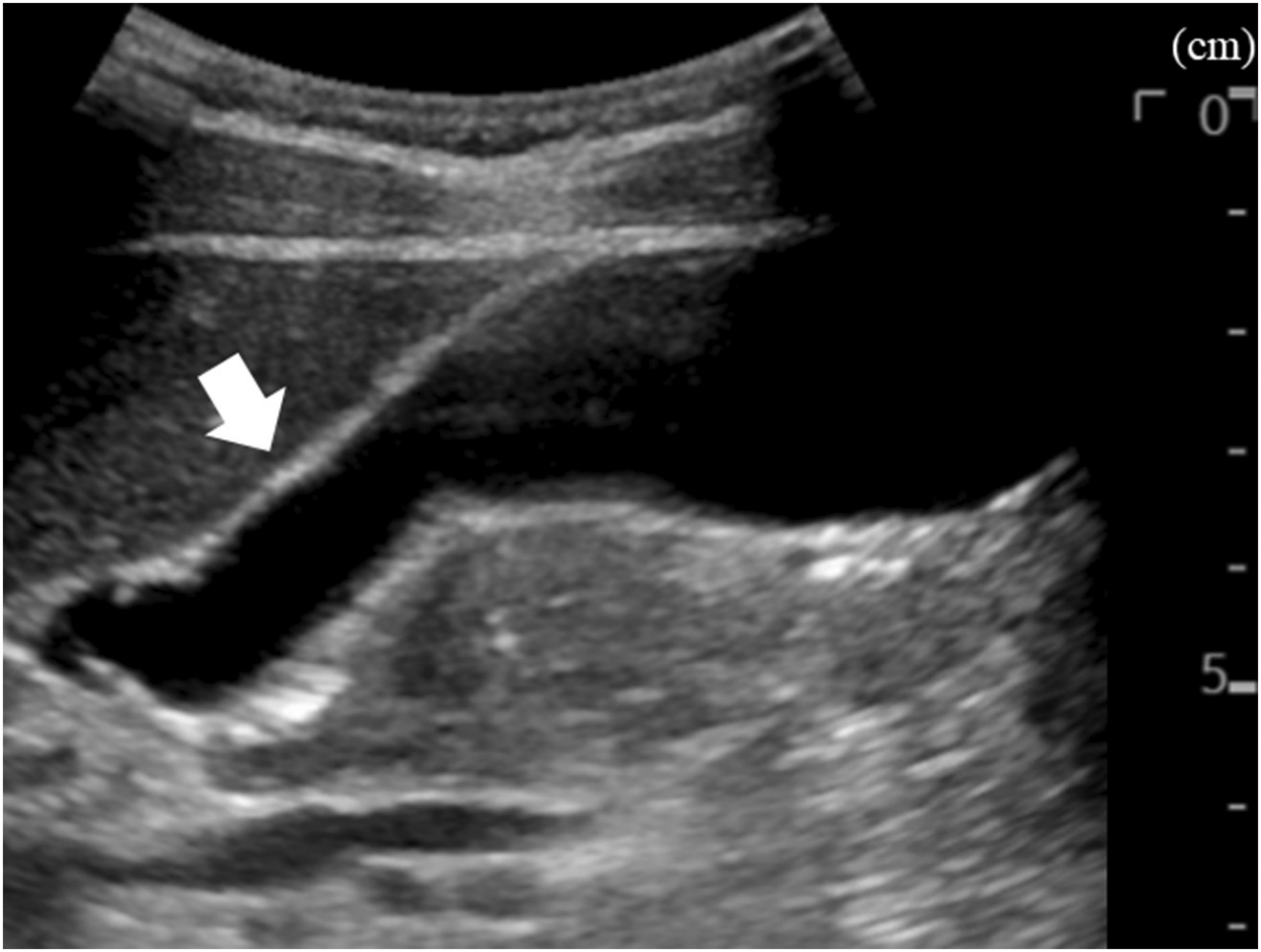
Findings of the gallbladder wall on abdominal ultrasonography 6 days after discharge. Abdominal ultrasonography 6 days after discharge showing improved gallbladder wall thickening, with a measurement of 1.5 mm (arrow).

The patient was a 35‐year‐old Japanese man who underwent an annual checkup, with an unremarkable medical history and no medications, and presented with headache and fever of 39.7°C 7 days before admission. Owing to worsening headache and persistent fever, he was admitted to our hospital. Physical examination revealed swelling of bilateral posterior cervical lymph nodes and tenderness in the abdominal right upper quadrant, without jaundice. Laboratory findings were as follows: white blood cell count: 8400/μL (reference range, 3300–8600), lymphocyte percentage: 73% (18.3%–47.5%), C‐reactive protein: 14,000 μg/L (0–1400), aspartate aminotransferase: 186 IU/L (13–30), alanine aminotransferase: 243 IU/L (10–42), γ‐glutamyl transpeptidase: 215 IU/L (13–64), and total bilirubin: 30.8 μmol/L (6.8–20.5). Abdominal ultrasonography revealed a thickened gallbladder wall measuring 10 mm (Figure 1). Chest and abdominal contrast‐enhanced computed tomography revealed a markedly thickened gallbladder wall measuring 12 mm, hepatosplenomegaly, and multiple enlarged cervical, bilateral axillary, and para‐aortic lymph nodes (Figure 2). On the 6th hospital day, the atypical lymphocyte percentage in the peripheral blood increased to 32%, with positive immunoglobulin‐M and ‐G antibodies associated with EBV, and negative Epstein–Barr nuclear antigen. He was diagnosed with AAC owing to EBV‐induced IM. With only watchful waiting (except for the use of celecoxib), his general condition and laboratory findings improved (Figure 3), and he was discharged on the 8th hospital day. Six days after discharge, he underwent abdominal ultrasonography, which revealed that the gallbladder wall thickening had improved and measured 1.5 mm (Figure 4).

## 3 DISCUSSION

EBV‐induced IM can cause virus‐like symptoms including fever, tonsillopharyngitis, lymphadenopathy, or hepatosplenomegaly.^2^ Although AAC can be commonly caused by severe infections or injuries, this is rare with EBV‐induced IM, with an incidence of <0.1%.^1^ The mechanisms of AAC formation are varied: bile stasis, increased calcified bile levels, decreased cholecystokinin‐induced gallbladder contraction, gallbladder wall ischemia, and direct viral invasion or secondary infection of the gallbladder.^3^ In EBV‐induced IM, hepatitis due to EBV and bile stasis due to EBV invasion into the gallbladder epithelial cell wall can cause gallbladder wall thickening.^3^ AAC due to severe infections or injuries requires cholecystectomy or percutaneous drainage. In comparison, AAC due to EBV‐induced IM usually does not require antibiotics or surgery. The major difference between the two types of AAC is the degree of gallbladder wall thickening. The former is associated with gallbladder wall thickening to ≥3.5 mm, and the latter is associated with marked gallbladder wall thickening, with an average measurement of 9.5 mm (range: 4.2–16.0 mm).^1^ In the present case, the gallbladder wall was markedly thickened to 10–12 mm on ultrasonography and abdominal contrast‐enhanced computed tomography.

## 4 CONCLUSION

In patients with virus‐like symptoms and marked thickening of the gallbladder wall, especially >9.5 mm thickness, it is important to suspect AAC due to EBV‐induced IM.

## AUTHOR CONTRIBUTIONS

**Masahiko Nakamura:** Conceptualization; resources; writing – original draft. **Shun Yamashita:** Conceptualization; supervision; writing – original draft. **Masaki Tago:** Supervision; writing – review and editing. **Shu‐ichi Yamashita:** Supervision; writing – review and editing.

## FUNDING INFORMATION

No specific grant was received for this work from any funding agency.

## CONFLICT OF INTEREST STATEMENT

The authors state that they have no conflicts of interest.

## ETHICS STATEMENT

This manuscript conforms to the 1995 provisions of the Declaration of Helsinki (as revised in Brazil 2013).

## CONSENT

Written informed consent was obtained from the patient to publish this report, in accordance with the journal's patient consent policy.

## Data Availability

The data that support the findings of this study are available from the corresponding author upon reasonable request.
